# Methionine Sulfoxide Reductase A (MsrA) Deficient *Mycoplasma genitalium* Shows Decreased Interactions with Host Cells

**DOI:** 10.1371/journal.pone.0036247

**Published:** 2012-04-30

**Authors:** Kishore Das, Georgina De la Garza, Shivani Maffi, Sankaralingam Saikolappan, Subramanian Dhandayuthapani

**Affiliations:** 1 Regional Academic Health Center, University of Texas Health Science Center at San Antonio, Edinburg, Texas, United States of America; 2 Department of Microbiology and Immunology, University of Texas Health Science Center at San Antonio, Edinburg, Texas, United States of America; 3 Department of Molecular Medicine, University of Texas Health Science Center at San Antonio, Edinburg, Texas, United States of America; University of California Merced, United States of America

## Abstract

*Mycoplasma genitalium* is an important sexually transmitted pathogen that affects both men and women. In genital-mucosal tissues, it initiates colonization of epithelial cells by attaching itself to host cells via several identified bacterial ligands and host cell surface receptors. We have previously shown that a mutant form of *M. genitalium* lacking methionine sulfoxide reductase A (MsrA), an antioxidant enzyme which converts oxidized methionine (Met(O)) into methionine (Met), shows decreased viability in infected animals. To gain more insights into the mechanisms by which MsrA controls *M. genitalium* virulence, we compared the wild-type *M. genitalium* strain (G37) with an *msrA* mutant (MS5) *strain* for their ability to interact with target cervical epithelial cell lines (HeLa and C33A) and THP-1 monocytic cells. Infection of epithelial cell lines with both strains revealed that MS5 was less cytotoxic to HeLa and C33A cell lines than the G37 strain. Also, the MS5 strain was more susceptible to phagocytosis by THP-1 cells than wild type strain (G37). Further, MS5 was less able to induce aggregation and differentiation in THP-1 cells than the wild type strain, as determined by carboxyfluorescein diacetate succinimidyl ester (CFSE) labeling of the cells, followed by counting of cells attached to the culture dish using image analysis. Finally, MS5 was observed to induce less proinflammatory cytokine TNF-α by THP-1 cells than wild type G37 strain. These results indicate that MsrA affects the virulence properties of *M. genitalium* by modulating its interaction with host cells.

## Introduction

*Mycoplasma genitalium* is a cell wall-less bacterium and a human pathogen that causes sexually transmitted diseases such as urethritis in males and cervicitis in females [1], [2], [3]. It has been implicated in female reproductive diseases such as endometritis, pelvic inflammatory diseases and others [4], [5], [6]. Increasing evidences suggest that it may also be a cofactor for HIV transmission [7]. *M. genitalium* initiates colonization of epithelial cells in genital-mucosal tissues by attaching itself to host cells surface [8]. It primarily uses surface proteins (adhesins) P140 (*MgpB*) and P32 [9], encoded by genes MG_191 and MG_318 respectively, for cell adherence. This process is assisted by a group of proteins called cytadherence accessory proteins that include several high molecular weight proteins (HMW) [8], [10], [11]. These proteins facilitate the translocation and positioning of adhesins on the surface to form the so called ‘attachment organelle’ which mediates the attachment process. In addition to attaching to host cells, *M. genitalium* has the ability to invade the host cells and persist there indefinitely [12], [13]. Recent in vitro studies have shown that lipid associated membrane proteins (LAMPs) from *M. genitalium* induce proinflammatory responses in monocyte derived macrophages which play a role in the clinical manifestations of the disease [14], [15], [16].

During host-pathogen interactions, mononuclear phagocytic cells (eg.macrophages) initiate the first line of defense against invading pathogens. These phagocytic cells have an array of antimicrobial responses which include generation of reactive oxygen species (ROS) and reactive nitrogen species (RNS) [17]. Phagocytes use two different pathways to produce the reactive species. While phagocyte oxidase (NOX2/gp91^phox^) produces superoxide (O_2_^·−^) [18], [19], inducible nitric oxide synthase (iNOS; NOS2) produces nitric oxide (NO). The superoxide (O_2_^·−^ ) undergoes a dismutation reaction or reacts with other compounds to produce hydrogen peroxide (H_2_O_2_) and reactive oxygen intermediates [20] such as HO^·−^, -OOH^·−^, etc. Likewise, reaction of NO with other compounds produces reactive nitrogen intermediates (RNI) such as HNO_2_, NO_2_^·−^. O_2_^·−^ and NO also reacts to produce the most potent peroxynitrite, (ONOO^·−^) [21], [22]. In addition to host derived ROS, some bacterial pathogens produce ROS as a consequence of aerobic metabolism. Regardless of the source, both ROIs and RNIs have the ability to damage macromolecules such as proteins, lipids, carbohydrates and nucleic acids. Bacteria use the antioxidants to detoxify ROIs and RNIs. Conventional antioxidants include enzymes like catalase-peroxidase (KatG), superoxide dismutase (SOD), alkyl hydroperoxide reductase (AhpR), organic hydroperoxide reductase (Ohr) and related enzymes. Interestingly, with the exception of Ohr, *M. genitalium*, lacks most of these antioxidant enzymes. This may be the result of its small genome. In *M. genitalium*, however, there are two copies of genes encoding Ohr (MG_427 and MG_454) in *M. genitalium*. We have recently determined the role of Ohr encoded by MG_454. This protein appears to defend against oxidative stress in in vitro experiments [23].

In addition to conventional antioxidants, methionine sulfoxide reductases (Msr), which specifically reduce the oxidized methionine (Met-O) to methionine (Met) through a reduction reaction involving thioredoxin, thioredoxin reductase and NADPH [24], also have roles in the detoxification of ROI. The oxidation of methionine leads to two different sulfoxides namely Met-*S*-O and Met-*R*-O, which are stereo-isomers. To reduce these sulfoxides, two enzymes, MsrA and MsrB, exist in most living organisms. In *M. genitalium* these enzymes are encoded by *MG_408* and *MG_448*. These two enzymes are unrelated structurally [25], [26], [27], [28] and studies have demonstrated that MsrA reduces Met-*S*-O and MsrB reduces Met-*R*-O [24], [27], [29], [30]. The organization of *msrA* and *msrB* varies in different bacterial species and four different types of organization have been observed. The different organizations include: a) *msrA* and *msrB* genes being located individually in different regions of the chromosome as separate transcription units, b) *msrA* and *msrB* genes located next to each other as separate genes but co-transcribed as a single transcription unit, c) *msr*A and *msrB* genes fused together as a single gene to produce a single protein with two domains, and d) *trx*, *msrA and msrB* genes fused together as single gene to produce a single protein with three domains. Interestingly, few bacteria have multiple copies of the genes encoding either *msrA* or *msrB* or both and few species completely lack genes coding for both enzymes [31]. In a subset of bacteria, Msr is encoded by genes that are present in both plasmid and chromosomal DNA [32].

Msr activity has been shown to be important in resisting oxidative stress in bacteria. However, exceptions have been observed in both A. *actinomycetemcomitans* [33] and *Mycobacterium tuberculosis* [34]. The absence of MsrA in most bacteria leads to increased susceptibility to oxidants in vitro [35], [36], [37], [38], [39], [40], [41], [42]. This phenotype could be reversed by complementation of the mutant strain with functional *msrA*. Bacteria deficient in MsrB also have a similar phenotype for oxidative stress, although there are few exceptions as with MsrA. In addition, MsrA has also been reported to be an important virulence factor in pathogenic bacteria because the absence of this protein affects a range of properties like adherence [36], [43], [44], [45], motility [38] biofilm formation [46], [47], intracellular survival [37] and in vivo survival [36], .

Previously, we have shown that MsrA was a virulence determinant in *M. genitalium*, as *M. genitalium* lacking MsrA was less able to adhere to sheep erythrocytes and to survive in hamsters [36].To gain more insights into the mechanisms by which MsrA affects virulence in *M. genitalium*, we compared the wild-type *M. genitalium* strain (G37) and *msrA* mutant (MS5) strain for their ability to interact with cervical epithelial cell lines (HeLa and C33A) and THP-1 monocytic cells. In studies related to bacterial pathogenesis, the routine approach has been to complement the mutant strains to determine the effect of particular gene products. Unfortunately, lack of integration and replicating plasmids poses severe restrictions in complementing *M. genitalium* mutant strains with other genes. However, we have used appropriate controls which include an *M. genitalium* strain, MGRE, which has a gentamicin resistant gene in an unrelated locus. We have shown that an *msrA* mutant strain has reduced ability to influence the physiology of target cervical epithelial cell lines (HeLa and C33A) and THP-1 monocytic cells. We also show that the MS5 strain is more susceptible to phagocytosis by THP-1 cells than wild type G-37 strain. Finally, we report here that MsrA is localized primarily in the cytosolic fraction of *M. genitalium*.

## Materials and Methods

### Bacterial Growth

*M. genitalium* G37, the wild type strain, was grown in 100 ml of SP-4 medium at 37°C in 150 cm^2^ tissue culture flasks (Corning, NY) until the color of medium changes to orange. *msrA* mutant *M. genitalium* strain (MS5) and *M. genitalium* strain MGRE, an uncharacterized strain that has integration of gentamicin resistance gene in one of the *mgpa* repetitive regions, were also cultured in SP-4 medium containing 50 µg/ml gentamicin.

### Cell lines and their culture

Human cell lines THP-1 (TIB-202), HeLa (CCL-2), C33A (HTB-31) and mouse cell line RAW 264.7 (TIB-71) were purchased from American Type Culture Collection (ATCC, Manassas, VA). THP-1 cells were cultured in RPMI medium with 10% FBS (HyClone, Logan, UT). HeLa, C33A and RAW 264.7 cells were cultured in Dulbecco's modified Eagle's medium (DMEM) with 10% FBS. Cultures in both media were grown at 37°C in a humid chamber with 5% CO_2_.

### Preparation of *M. genitalium* strains for infection

Surface adherent mycoplasmas (G37, MS5 and MGRE) were washed four times with phosphate-buffered saline (PBS; pH 7.2), scraped with cell scraper (39 cm handle/3 cm blade; Corning, NY) and collected by centrifugation (20,000×g, 20 min, 4°C) using a Sorvall RC 5B centrifuge. The bacterial pellets were resuspended in PBS and passed first though 18G needles and then through 23G needles to disperse bacterial clumps. The suspensions were diluted in PBS to *A*_600_ = 1.0. This was further diluted in appropriate volume of PBS to infect cell lines with different multiplicity of infection.

### Cytotoxic assay

A cytotoxic assay based on sulforhodamine B (SRB) was adapted from Vichai and Kirtikara [49]. In brief, HeLa and C33A cells were plated in triplicate on 96 well plates (5,000 cells/well) for 24 h, they were then infected with G37 and MS5 bacteria at different multiplicity of infection. Cells were incubated at 37°C in 5% CO_2_ for 12 h, followed by fixing for 1 h with 10% cold trichloroacetic acid (TCA). Plates were then washed five times in water, air-dried and stained with 0.057% SRB for 30 min. The plates were washed again four times with 1% acetic acid, air-dried, and bound SRB was dissolved in 10 mM unbuffered Tris base (pH 10.5). The absorbance at *A*_510_ was determined using a SpectraMax M5 microplate reader (Molecular Devices, Sunnyvale, CA). The percent survival was calculated based on the absorbance values relative to untreated samples.

Integrity of cell lines after infection with *M. genitalium* strains G37, MS5, MGRE and heat killed *M. genitalium* (HKG37) was assessed using an Olympus FV1000 confocal laser scanning microscope and by capturing Differential Interference Contrast (DIC) images with 20× objective (NA 0.75) with 488 nm laser.

### CFSE labeling of THP-1 cells

Carboxyfluorescein diacetate succinimidyl ester (CFSE) labeling of THP-1 cells was done as described by Clanchy et al. [50]. Briefly, THP-1 cells were pelleted by centrifugation at 125×g for 6 min and resuspended to10^6^ cells/ml in PBS. CFSE (1 µM) was added and the suspension was incubated at room temperature for 10 min. The reaction was quenched by addition of RPMI medium containing 5% serum followed by washing with PBS containing 1% FBS. CFSE labeled THP-1 cells were seeded in 4 well 1.5 German cover glass chambers (Nunc, Rochester, NY) at 0.5×10^5^ cells per well in RPMI medium and infected with *M. genitalium* strains G37, MS5, MGRE and HKG37 (MOI 1∶5) for 1 h. The chambers were washed twice with PBS to remove non-adherent cells, and images of adherent cells were acquired using Olympus FV1000 confocal laser scanning microscope with 10× objective (NA 0.40) and 488 nm laser. The number of labeled cells in each image were counted using the particle plugin of NIH Image J software. Average cell numbers from five different optical fields and from three independent experiments were used for determining the number of adherent mononuclear cells in each infection.

### Phagocytic assay

Phagocytic assays were done using color change assay based on the ability of surviving bacteria to reduce the chemical MTS to purple formazan using CellTiter 96 Aqueous One Cell Proliferation Assay kit (Promega). Briefly, 1×10^5^ THP-1 cells were seeded on 96 well plates and differentiated by 100 nM of Phorbol-12-myristate-13-acetate (PMA) for 48 h in RPMI medium containing 10% serum. The differentiated THP-1 cells were replaced with 100 µl of SP-4 and infected with G37 or MS5 or MGRE (MOI 1∶10) and incubated at 37°C in 5% CO_2_ for 1 h. Control wells contained equal amount of bacteria in 100 µl of SP-4 medium. After 1 h of incubation, the media from the infected wells were transferred to wells in a fresh 96 well plate. To remove the adherent bacteria fully, the wells from the infected plate were rinsed twice with 50 µl of SP-4 and collected in corresponding wells in the new plate. To maintain equal volumes, 100 µl of SP-4 medium was added to all the control wells. Next, 40 µl of CellTiter 96 Aqueous reagent was added to all wells, incubated for 1 h at 37°C and absorbance was determined at 490 nm using a SpectraMax M5 microplate reader (Molecular Devices, Sunnyvale, CA). Absorbance readings obtained for vehicle control wells (PBS) were used as blank. The difference in absorbance between the experimental wells (infected) and control wells were calculated to determine the amount of bacteria phagocytosed by THP-1 cells. To confirm the color change assay, phagocytosis of G37 or MS5 or MGRE was visualized by staining the bacteria with Fluorescein isothiocyanate isomer I (FITC; Sigma-Aldrich, St. Louis, MO) before infection, as described before [51]. For this experiment, THP-1 cells (1×10^5^ ) were placed on a 4 well 1.5 German cover glass chamber (Nunc, Rochester, NY) and differentiated with PMA (100 nM) for 48 h. Bacterial cells were labeled with FITC by directly resuspending the pelleted bacteria in a solution containing FITC (FITC 0.1 mg/ml in 0.1 M NaHCO_3_, pH 9.0) for 1 h at 25°C [52]. Labeled bacterial cells were washed four times with PBS and resuspended in PBS. The bacterial cells were used to infect the differentiated THP-1 cells by incubating for 1 h at 37°C with 5%CO_2_. After adding ethidium bromide (50 µg/ml), the infected cells were washed three times gently with PBS. Images of the cells were captured using an Olympus FV 1000 confocal scanning microscope with 488 nm laser and standardized settings (gain, laser, zoom) across all experimental groups.

### Reactive oxygen species (ROS) detection

Generation of Reactive Oxygen Species (ROS) was determined using the fluorescent probe 2′, 7′-Dichlorodihydrofluorescein diacetate (DCF-DA). RAW 264.7 cells (1×10^5^) cultured in 4 well 1.5 German cover glass chamber (Nunc, Rochester, NY) for 12 h, were infected with G37, MS5, MGRE and HKG37 (MOI 1∶10) for 1 h at 37°C and 5% CO_2_. After infection, cells were washed twice with 1× PBS and ROS generation was detected by adding 1 µM DCF-DA for 30 min at 37°C. Images were captured using a FV 1000 confocal scanning microscope at 488 nm laser setting as described above. For reproducibility and comparison, all experimental conditions and microscope settings were kept identical for all the experiments. Image processing and data analysis were done with NIH Image-J software. Fluorescence values from captured images of 10 different fields and from three independent experiments were calculated and the results expressed as arbitrary fluorescence units.

### Cytokine assays

Pro-inflammatory cytokines TNF-α and IL1-β released by THP-1 and HeLa cells in response to infection by *M. genitalium* strains G37, MS5 and heat killed G37 (HKG37) cells were determined by ELISA. First, THP-1 cells (1×10^5^) and HeLa cells ( 1×10^4^) were plated in 96 well plates and infected with mycoplasma strains mentioned above (MOI 1∶10). Supernatants, after 1 h and 12 h post infection for THP-1 cells and HeLa cells, respectively, were collected and centrifuged briefly to settle any floating cell debris. The supernatants were diluted (1∶4) using PBS and TNF-α and IL-1β concentrations were determined using an ELISA kit (Bioscience, eBioscience.com) following manufacturer's protocol.

### Preparation of membrane and cytosolic fractions

Cell membranes and cytosolic fractions of mycoplasma strains were prepared by osmotic lysis as described by Razin [53]. Briefly *M. genitalium* G37 and MS5 cell pellets were resuspended in 2 ml of 0.25 M NaCl. Osmotic lysis of the cells was done by addition of 4–6 ml of deionized water preheated to 37°C followed by incubating for 15 min at room temperature. Membranes were collected by centrifugation 34,000×g for 30 min using a Sorvall Discovery M120SE centrifuge (Kendro, Asheville, NC). Membranes were washed twice in PBS and dissolved in β-buffer (0.15 M NaCl, 0.01 M β-mercaptoethanol, 0.05 M Tris-HCl, pH 7.4 diluted to 1∶20 in deionized water). The supernatant (cytosol) was concentrated by ultrafiltration using Amicon® Ultra-4 Centrifugal Filter Units with 3 kDa cut off (Millipore, Milford, MA,) and protein concentrations of all fractions were determined using Pierce BCA Protein Assay Kit (Thermo Scientific, Rockford, IL).

### Immunoblot analysis

SDS-PAGE and Western blots were performed following standard protocols [54]. Proteins (20 µg/lane) from whole cell lysate, membrane and cytosol fractions of G37 and MS5 were resolved using NuPAGE 12% Bis-Tris Gel (1.0 mm×10 well; Invitrogen, Carlsbad, CA). Proteins were transferred to Whatman Protran nitrocellulose membranes (Whatman, Dassel, Germany), blocked with 5% skim milk and probed with anti-*M. pneumoniae* MsrA rabbit serum [36] [1∶1,000 in Tris-buffered saline containing 0.1% Tween-20 (TBST)] or anti-*M. pneumoniae* elongation factor G (EF-G) rabbit antiserum (1∶2000 in TBST) [55]. After washing three times for 5 min each with TBST, membranes were incubated at room temperature for 1 h with horseradish peroxidase (HP)-conjugated goat anti-rabbit IgG (Sigma Aldrich, St. Louis, MO) at a concentration of 1∶10,000 in TBST. The blots were developed with Pierce ECL Western blotting substrate (Thermo Scientific, Rockford, IL) and the signals were captured with BioMax Light Film (KODAK, Rochester, NY).

### Immunofluorescence staining of MsrA

To examine the localization of MsrA, immuno-staining protocol was used as described by Wayne et al. [56]. Briefly mycoplasma strains (G37 and MS5) cells were pelleted as described above and were fixed in 2% paraformaldehyde for 30 min, permeabilized with 0.1% Triton X-100 and 0.1% Tween-20 in PBS for 30 s, followed by blocking with PBS containing 5% BSA and 5% normal serum. Cells were suspended using a 23G needle followed by overnight incubation at 4°C with anti-MsrA rabbit serum. The bacterial cells were washed three times with PBS followed by staining with Fluorescein anti-rabbit IgG (Vector, Burlingame, CA) for 2 h in room temperature. The cells were washed three additional times with sterile PBS, suspended uniformly using a 23G needle and mounted on a glass slide. Images were captured with an Olympus FV 1000 confocal scanning microscope using a 60× objective (NA 1.42) and 488 nm Argon laser settings with an electronic zoom of 5×. Parallel experiments were done with non-permeabilized cells as controls.

### Statistics

Paired *t*-test was performed using graphpad prism software.

## Results

### *msrA* mutant strain of *M. genitalium* is less cytotoxic

**Figure 1:**
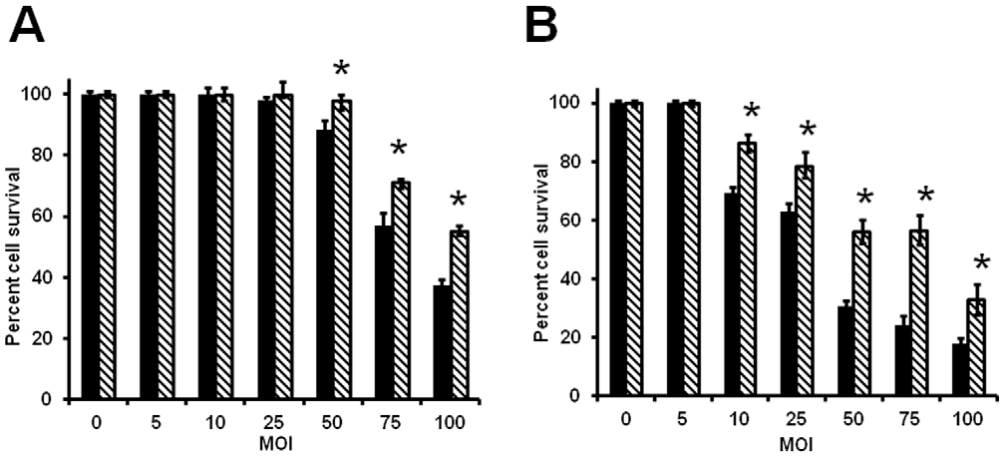
Cytotoxic effect of *M. genitalium* strains on cervical epithelial cells based on cell survival assay. HeLa (**A**) and C33A (**B**) epithelial cells infected with *M. genitalium* G37 (wild type) and MS5 (*msrA* mutant) strains at different multiplicity of infections and cells survived was determined by SRB (sulforhodamine B) assays. Solid bars in figures **A** and **B** represent *M. genitalium* wild type strain G37. Bars with downward stripes in figures **A** and **B** represent *M. genitalium msrA* mutant strain MS5. Both strains were tested at various MOI (1∶0 −1∶100). * = p≤0.05 percent cell survival of MS5 strain is higher vs G37 strain. Results represent Mean ± SD of three independent experiments.

Cytotoxicity is a virulence mechanism in few pathogenic mycoplasmas including *Mycoplasma pneumoniae* [57], [58]. To understand if the deletion of *msrA* gene has any effect on the cytotoxicity of *M. genitalium*, we compared the cytotoxic effects of *M. genitalium* wild type (G37) and *msrA* mutant (MS5) strains of *M. genitalium* by infecting HeLa and C33A epithelial cell lines with multiple MOIs and measuring the SRB signal from the culture. Both cell lines showed decreased cell survival due to cytotoxicity by *M. genitalium* strains, although they differed in their responses (Fig. 1A and 1B). HeLa cells displayed little cell death even at a high MOI of 1∶50 and a significant decline in cell survival was noticed only with MOI of 1∶75 and greater. In contrast, C33A cells showed a decline in cell survival at lower MOI of 1∶10. Increased levels of cell deaths were observed with increased levels of infections in both cell lines, although a significant portion of cells were still alive even after infecting these cell lines with an MOI of 1∶100. However, comparison of cytotoxic effects between G37 and MS5 strains revealed that the latter had significantly lower cytotoxic effects on both cell lines.

**Figure 2:**
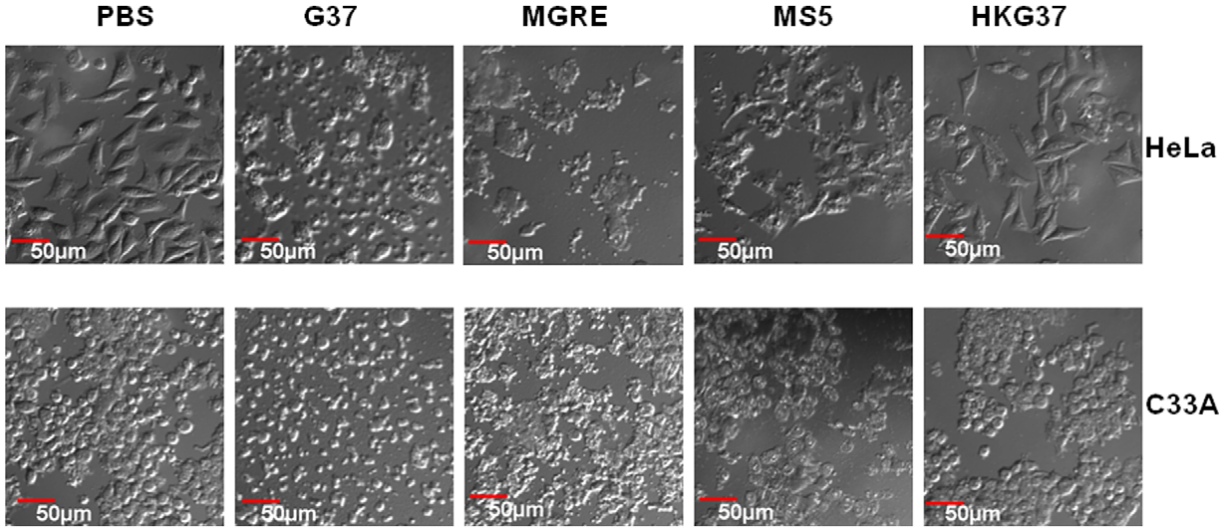
Microscopic observation of cytotoxic effect of *M. genitalium* strains on cervical epithelial cells. HeLa and C33A epithelial cells were infected with *M. genitalium* G37 and MS5 strains and analyzed using differential interference contrast at 488 nm in a confocal laser scanning microscope with 20× objective. PBS indicates uninfected control cells; G37, MGRE, MS5 and HKG37 indicate infection of cells with *M. genitalium* wild type G37 strain, control strain MGRE, *msrA* mutant strain MS5 and heat killed G37 bacteria, respectively.

To confirm the results obtained with the SRB assays, the cytotoxicity of these strains were visually analyzed with a microscope. As can be seen in Fig. 2, no cell lysis was observed with the cells treated with PBS (control) or cells infected with heat killed G37 strain. In contrast, cells infected with live G37, MGRE and MS5 strains show significant lysis of the cells. Cell lysis by MS5 is only moderate as compared to G37 strain, thus reflecting the results observed with the SRB assay. These data demonstrate that MsrA is an important component required for the cytotoxic effect of *M. genitalium*. To investigate whether the cytotoxic effect of *M. genitalium* results in necrotic or apoptotic cell death, HeLa cells infected with G37strain were stained using the Apoptotic/Necrotic/Healthy Cells Detection kit (PromoKine, Heidelberg, Germany). This differentiates apoptotic and necrotic cell death by green and red fluorescence, respectively. Confocal microscopic observation of the cells revealed (data not shown) that 99% of the dead cells stained with red fluorescence, indicating that cell death due to *M. genitalium* cytotoxicty was primarily through a necrotic pathway.

### *msrA* mutant strain of *M. genitalium* is more susceptible to phagocytosis

**Figure 3:**
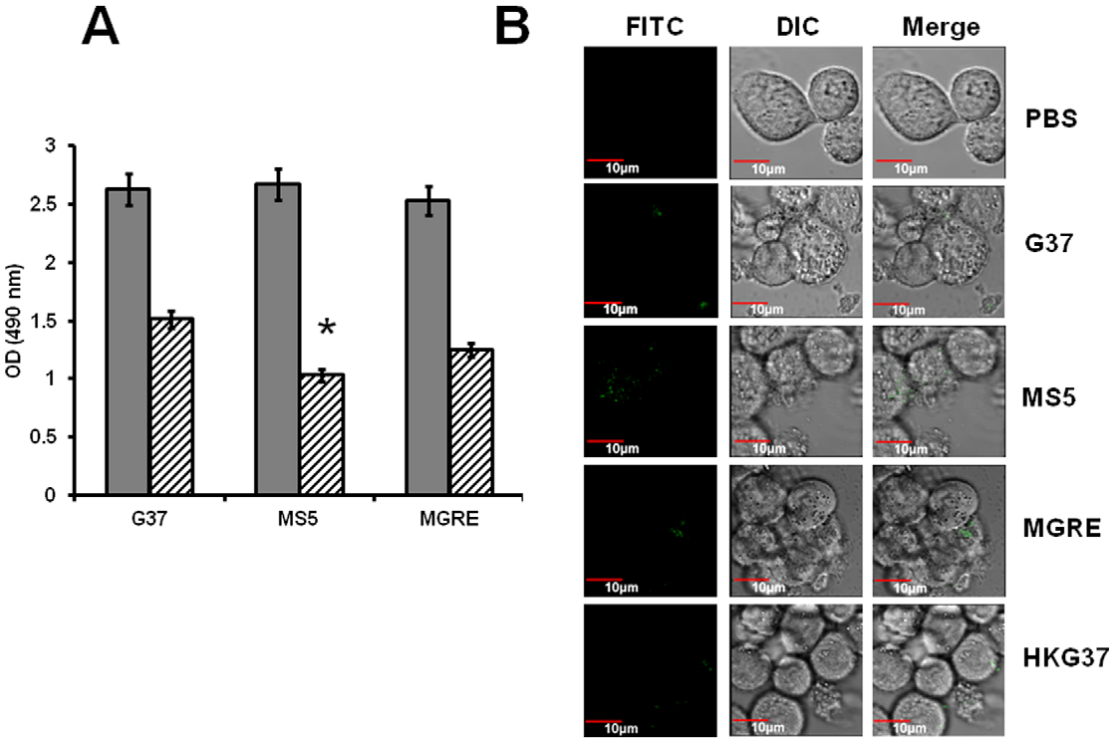
Phagocytosis of *M. genitalium* strains by THP-1 cells. **A. Determination of phagocytosis by color change method.** Phagocytosis of *M. genitalium* strains by THP-1 cells were determined by a change in color after adding MTS solution (Promega) as described under Materials and Methods section. The solid bars indicate absorbance (*A*_490_) of the control wells (Mycoplasmas without THP-1 cells) and striped bars represent absorbance (*A*_490_) of the experimental wells (THP-1 cells infected with mycoplasmas). Results represent Mean ± SD from three independent experiments. G37, MGRE and MS5 indicate infection of cells with *M. genitalium* wild type G37 strain, control strain MGRE and *msrA* mutant strain MS5, respectively. * = p≤0.05 vs wild type G37. **B. Visualization of phagocytosed** ***M. genitalium*** **G37, MGRE and MS5 strains.** G37, MGRE and MS5 bacteria were labeled with FITC as described in Material and Methods. FITC and DIC represent fluorescence and differential interference contrast of the same field. Merge represents overlay of FITC and DIC. PBS indicate uninfected control cells; G37, MGRE and MS5 indicate infection of cells with *M. genitalium* wild type G37 strain, control strain MGRE and *msrA* mutant strain MS5, respectively.

Phagocytosis of invading pathogens by host macrophages is an important event in host-pathogen interactions. A recent study reported the dynamics of *M. genitalium* phagocytosis by human monocyte derived macrophages [59]. To assess if there is a difference between G37 and MS5 strains in phagocytosis by macrophages, PMA differentiated THP-1 cells were exposed to these mycoplasma strains and the uptake was assessed using a color change assay. The results indicate that after 1 h postinfection, significantly more MS5 bacteria were phagocytosed than either G37 or MGRE strains (Fig. 3A). To further confirm this observation, FITC labeled strains of *M. genitalium* were allowed to be phagocytosed by THP-1 cells. Confocal analysis (Fig. 3B) of the THP-1 cells revealed that fluorescent bacteria were higher in the phagosomes of THP-1 cells infected with MS5 strain than phagosomes of THP-1 cells infected with G37 and MGRE strains, confirming the results from color change assays. These observations suggest that the absence of MsrA leads to enhanced uptake of *M. genitalium* by THP-1 cells.

### *msrA* mutant strains of *M. genitalium* induces high ROS in RAW264.7 cells

**Figure 4:**
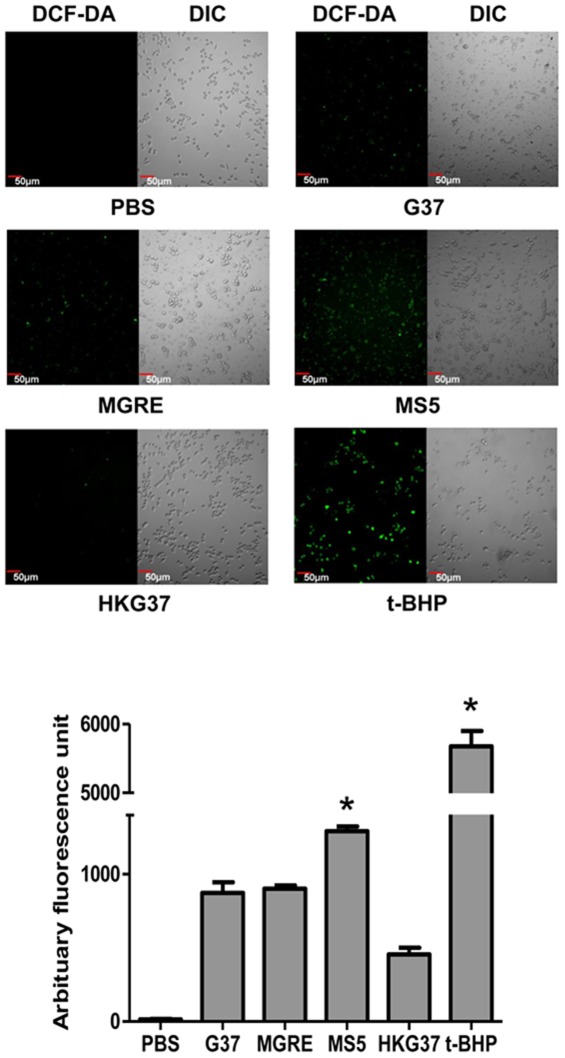
Generation of Reactive Oxygen Species (ROS) by phagocytic RAW264.7 cells upon infection with *M. genitalium* strains. **A. Confocal images of RAW264.7 cells showing ROS generation.** RAW264.7 cells were infected with G37, MS5, MGRE or treated with heat killed G37 (MOI 1∶10) and generation of ROS was detected by addition of DCF-DA. Images were captured using an Olympus confocal laser scanning microscope with 488 nm laser. DCF-DA and DIC indicate fluorescence of DCF-DA and differential interference contrast of the same field; G37, MS5 and MGRE represent cells infected with *M. genitalium* wild type, *msrA* mutant and control strains. HKG37 represents cells treated with heat killed wild type *M. genitalium*. *t*-BHP represents ROS induced with 1 µM *t*-butyl hydroperoxide for 30 min. **B. Graphical representation of ROS generated by RAW264.7 cells.** Each bar represents ROS generated by RAW264.7 cells in response to infection/induction. Images were captured using a confocal laser scanning microscope. Total fluorescence counts were determined from images using NIH image J software from ten different fields and three independent experiments. Arbitrary fluorescence units for each infection are given as Mean ± SD. * = p≤0.05 vs wild type G37. Labels are as described in “**A**”.

Macrophages generate reactive oxygen species (ROS) to combat microbial pathogens. Observed higher uptake of MS5 (*msrA* mutant) than G37 strain by THP-1 cells led us to hypothesize that MS5 ingested THP-1 cells might generate more ROS than wild type G37 ingested THP-1 cells. To test this hypothesis, THP-1 cells were infected with the *M. genitalium* MS5 strain and other control strains and the generation of ROS was determined by using fluorescent ROS detector DCF-DA. Due to high ROS background of THP-1 cells, it was difficult to determine ROS generated due to infection. Therefore, a RAW264.7 mouse macrophage cell line, which was reported to have less indigenous ROS, was used [60]. Consistent with our hypothesis, RAW264.7 cells infected with MS5 strain and stained with DCF-DA displayed significantly higher levels of fluorescence, than cells infected with G37 strain or MGRE strain (Fig. 4A and B), indicating that more ROS was produced by cells infected with MS5. This reconfirmed that *msrA* mutant MS5 strain is more susceptible to phagocytosis and consequently to oxidative killing by phagocytes. Nonetheless, the possibility exists that a portion of the ROS detected in MS5 infected cells might include ROS generated by mycoplasmas.

### *msrA* mutant strain of *M. genitalium* is less efficient in differentiating THP-1 cells

**Figure 5:**
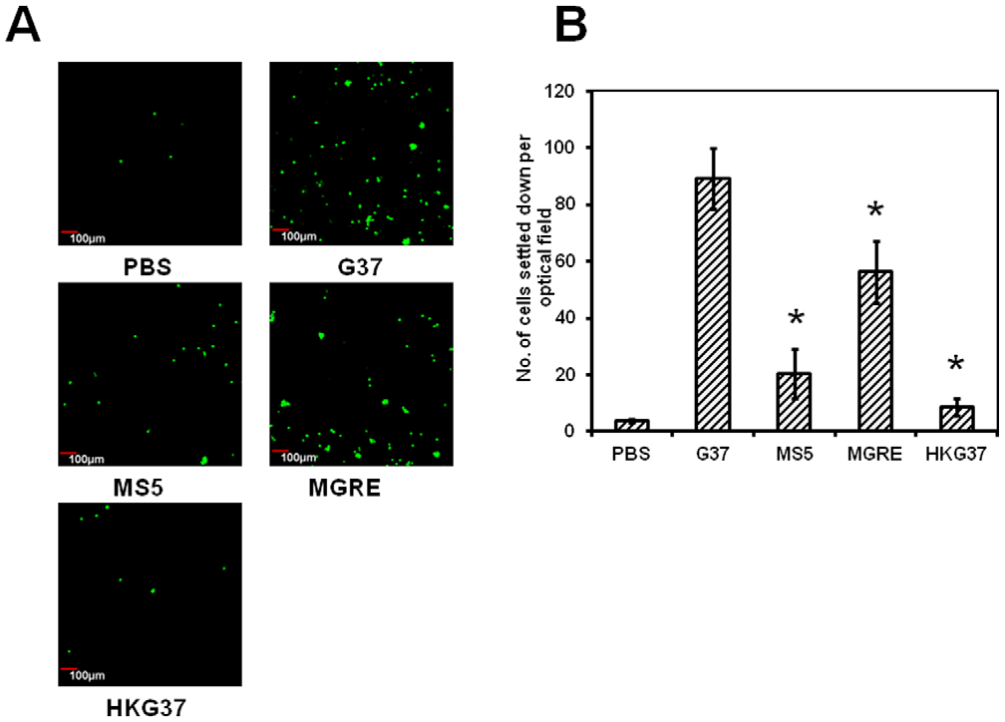
Differentiation of THP-1 cells by *M. genitalium* strains. **A. Adherent THP-1 cells showing fluorescence.** CFSE labeled THP-1 cells were infected with *M. genitalium* strains (MOI 1∶5). Images of adherent cells were acquired using confocal laser scanning microscope with 10× objective and 488 nm laser. G37, MS5 and MGRE are wild type, *msrA* mutant and control *M. genitalium* strains respectively. HKG37 represents heat killed bacteria of wild type *M. genitalium*. **B. Graph showing the amount of adherent cells for each infection.** The number of labeled cells in each image were counted using the particle plugin of Image J software. Average cell numbers from five different optical fields and from three independent experiments were used for determining the number of adherent mononuclear cells in each infection. Labels are as described in “**A**”. * = p≤0.05 vs wild type G37 strain.

It has been well established that *Mycoplasma fermentens* has the ability to differentiate mononuclear cells [61]. In this study, the ability of *M. genitalium* to differentiate mononuclear cells by infecting CFSE stained undifferentiated THP-1 cells with wild type *M. genitalium* strain G37 was investigated. THP-1 cells showed differentiation and as a result the cells adhered to the bottom of the flasks and culture slides, similar to that seen with cells differentiated by PMA. To understand if *msrA* mutant strain (MS5) has similar ability to differentiate mononuclear cells, THP-1 cells were infected with this strain and the cells were assessed for their adherence. As shown in Fig. 5A and B, relatively lower numbers of THP-1 cells infected with MS5 strain adhered to the slide surface as compared to G37 infected cells. It was also observed that the ability of the MS5 strain in differentiating THP-1 cells was lower than the level of MGRE strain but higher than the level of the heat killed G37 bacteria. These results suggest that the absence of MsrA protein affects some components on *M. genitalium* that are required for the induction of differentiation of mononuclear cells.

### *msrA* mutant strain of *M. genitalium* induces less TNFα

**Figure 6:**
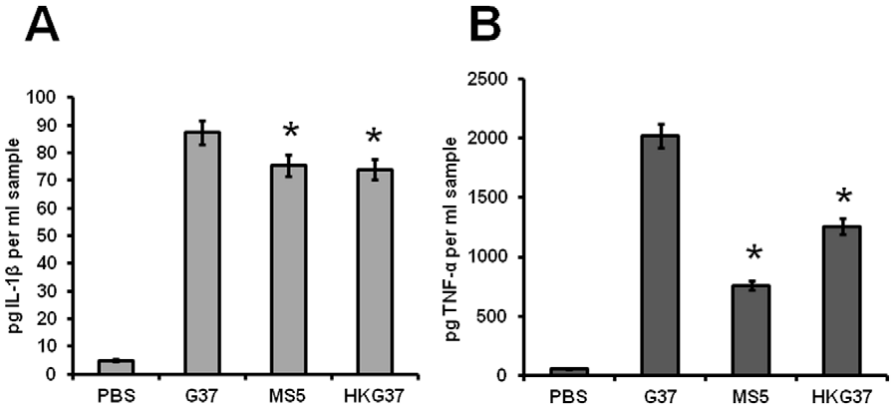
Cytokines released by THP-1 cells after infection with mycoplasma strains. Supernatants from THP-1 cells infected with mycoplasma strains (MOI 1∶10) were collected and IL-1β and TNF-α concentrations were determined using an ELISA kit from eBioscience. **A.** Release of IL-1β by THP-1 cells and **B.** Release of TNF-α by THP-1 cells. PBS, phosphate buffered saline control; G37 and MS5 are wild type *msrA* mutant *M. genitalium* strains respectively; HKG37 represents heat killed wild type *M. genitalium*. * = p≤0.05 vs wild type G37.

*M. genitalium* causes inflammation in genital tissues by inducing proinflammatory cytokines. Since *msrA* mutant (MS5) strain exhibited less virulent properties than the wild type (G37) strain, we tested for the differences between the two strains in inducing proinflammatory cytokines IL-1β and TNF-α. To determine this, both HeLa and THP-1 cell lines were infected with G37 and MS5. Heat killed G37 was used as a control. ELISA assays of cytokines released by the cells into the medium revealed that *M. genitalium* strains induced only negligible amount of IL-1β and TNF-α in HeLa cells after 12 h postinfection (data not shown). In contrast, all strains induced significant amounts of IL-β and TNF-α in THP-1 cells in an infection time of 1 h (Fig. 6). Although there was a moderate difference between G37 and MS5 strains in the induction of IL-1β by THP-1 cells (Fig. 6A), MS5 strain exhibited significantly lower induction of TNF-α (50%) than wild type strain (G37) (Fig. 6B). In addition to live G37 and MS5 strains, significant induction of both cytokines were observed by heat killed G37 in THP-1 cells, which suggests that certain components of *M. genitalium* may be sufficient to induce immune response.

### MsrA is primarily localized to the cytosol

**Figure 7:**
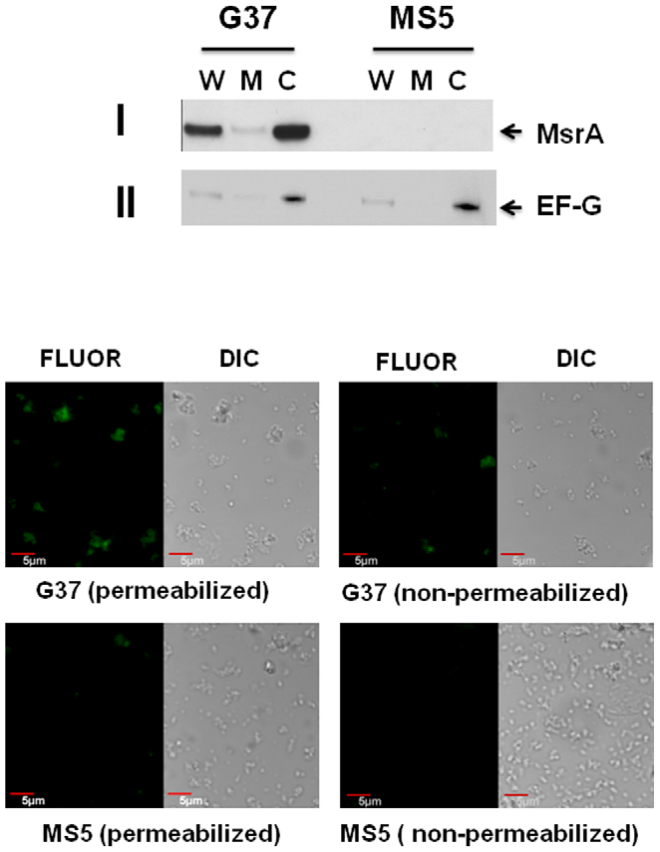
Localization of MsrA in *M. genitalium*. **A. Immunoblot analysis.** Cytosol and membrane fractions were prepared from *M. genitalium* strains, separated by SDS-PAGE, transferred to nitrocellulose membrane and probed with anti-MsrA (**I**) and anti-EF-G (**II**). G37 and MS5 represent *M. genitalium* wild type and *msrA* mutant strains, respectively. W, M and C indicate whole, membrane and cytosol fractions of *M. genitalium*. MsrA indicates the bands reacting with anti-MsrA antiserum. EF-G indicates the bands reacting with anti-EFG antiserum. **B. Immunohistochemical localization of MsrA.** *M. genitalium* (G37 and MS5) cells were suspended uniformly, fixed with paraformaldehyde and permeabilized with Triton X-100 and Tween-20 or not permeabilized. This was followed by incubation in anti-MsrA antiserum and fluorescein conjugated anti-rabbit IgG secondary antibody. Images were captured with a FV 1000 confocal laser scanning microscope using 60× objective with 5× electronic zoom. FLUOR and DIC represent fluorescent and differential interference contrast images of the same field. G37, *M. genitalium* wild type strain; MS5, *M. genitalium msrA* mutant strain.

Previous studies have demonstrated that MsrA is localized both in the cytosol and membrane fractions of bacteria [33], [35]. To determine the location of MsrA within *M. genitalium*, we prepared cytosol and membrane fractions from G37 and MS5 strains and probed these fractions with anti-MsrA antiserum (Fig. 7A). This antiserum reacted with MsrA and two cross reactive proteins (CRPs) in the wild type strain and only CRPs in the *msrA* mutant strain. The CRPs were found to be due to the reaction of second antibody with two *M. genitalium* proteins; hence they are not shown here. The results (Fig. 7A-I), however, revealed that both membrane and cytosolic fractions had MsrA but the amount of MsrA in the cytosolic fraction was several fold higher than the membrane fraction. To test whether this small amount of MsrA with membrane fraction was due to its association with membrane, we probed the same blot with anti-elongation factor G (EF-G; MP604) antiserum of *M. pneumoniae*, a marker for cytosolic protein [55], [62] that is 95% identical to EF-G (MG_089) of *M. genitalium*. Results (Fig. 7A-II) revealed moderately intense, less intense and intense bands for the whole, membrane and cytosolic fractions, respectively, in both strains. Repeated analysis resulted in similar pattern of reactivity for MsrA and EF-G in both cytosolic and the membrane fractions. There appeared to be no problem with the method used for the preparations of membrane and cytosolic proteins, since SDS-PAGE (Fig. S1) separated proteins showed distinct patterns for cytosolic and membrane proteins. It is not clear whether a portion of both proteins are associated with the membrane of this species or these proteins associate themselves to the membrane fractions during the preparation of membranes. However, since MsrA predominates in the cytosolic fraction like the cytosolic marker EF-G, we presume that MsrA is primarily a cytosolic protein in *M. genitalium*.

In addition to biochemical analysis, immunohistochemistry was also performed to confirm the localization of MsrA by using anti-MsrA antiserum and fluorescein conjugated second antibody (Fig. 7B). In this experiment, a detergent was used to permeabilize *M. genitalium* G37 and MS5 bacteria. Confocal analysis revealed that detergent treated G37 strain had significantly enhanced fluorescence as compared to untreated bacteria. In contrast, very limited fluorescence in the detergent bacteria and no fluorescence in the untreated bacteria, respectively, were seen in the *msrA* mutant MS5 strain. These results reinforce the observation that MsrA is primarily a cytosolic protein and that antibodies cross react with other proteins of *M. genitalium* as observed in Western analysis.

## Discussion

In this study we have investigated the effects of MsrA protein on *M. genitalium*'s interactions with host cells using *msrA* mutant and wild type strains. We observed that a loss of MsrA affects the ability of *M. genitalium* to cause cytotoxicity in host cells. Cytotoxicity is an important virulence mechanism of some pathogenic mycoplasmas that infect human and animal hosts, and one of the major components that induce cytotoxicity appears to be the H_2_O_2_ released by these species [57], [58]. Interestingly, generation of H_2_O_2_ in these species is linked to oxidation of glycerol by glycerol 3-phosphate oxidase [63]. This is evident from the disruption of the *glpD* gene in *M. pneumoniae* which codes for glycerol 3-phosphate oxidase [64]. It has been shown that a *glpD* mutant of *M. pneumoniae* is less able to produce H_2_O_2_ and as a consequence is less efficient in producing cytotoxicity [64], thus indicating a direct relationship between production of H_2_O_2_ and cytotoxicity. In addition to glycerol 3-phosphate oxidase, the ABC transport system is also implicated in the production of H_2_O_2_. This system is critical for transport of glycerol and strains of *M. mycoides* having deletion in *ABC* locus were found to have reduced cytotoxicity [65]. Extensive studies in this species reveal that release of H_2_O_2_ alone is not sufficient enough to cause cytotoxicity but the released H_2_O_2_ by mycoplasma also needs to be translocated to the host cells [66]. This indicates that a close contact between mycoplasma and host cells is required for cytotoxicity in which adherence plays a major role. In this context, we have previously reported [36] that *msrA* mutant *M. genitalium* adheres poorly with red blood cells and the reason for the reduced cytotoxicity by this strain may be the poor adherence. Alternatively, the possibility exists that absence of MsrA affects other sites related to production or translocation of H_2_O_2_ in the *msrA* mutant *M. genitalium*, thus leading to reduced cytotoxicty. It may be noted that GlpD and ABC transporter proteins are highly conserved in *M. genitalium* [67].

Although *M. genitalium* is able to survive intracellularly within cervical epithelial cells [12], [13], [68], its ability to survive within phagocytes is limited. A recent study demonstrated that human monocyte derived macrophages (MDM) phagocytose *M. genitalium* within five minutes of post infection and digest all ingested bacteria within six hours post infection [59], suggesting that *M. genitalium* is susceptible to phagocytosis mediated killing. Thus, MsrA of *M. genitalium* is less likely to play a significant role in defending ROS generated by macrophages. Nevertheless, the observation that the absence of MsrA in *M. genitalium* significantly increases the phagocytosis by THP-1 cells, as compared to wild type control, is very striking. Detection of significantly higher levels of ROS in *msrA* mutant ingested macrophages than wild type ingested macrophages provides additional support for the increased level of phagocyotsis by this strain. Since phagocytosis is a process based upon ligand–receptor interactions, the altered phagocytosis may indicate that there is a level of alteration of surface molecules that mediates binding to phagocytic cells in the *msrA* mutant strain. This is a possibility, because alterations in surface ligands/receptors of pathogens that lack MsrA have already been reported [43], [44]. Whereas *S. pneumoniae* lacking MsrA had a significant alteration in the ligand that binds GalNAcβ1-4Gal receptors of eukaryotic cells, enteropathogenic *E. coli* lacking MsrA had alterations in the ligand that binds with the mannose receptor [43]. Conversely, the receptors that bind with ECM molecules, like collagen, laminin and fibronectin, have also been found to be affected in *Streptococcus gordonii* that lack MsrA [44].

The observation that *M. genitalium* differentiates THP-1 cells phenotypically more or less similar to the action of PMA (phorbol 12-myristate 13 acetate) on these cells is interesting. To our knowledge, this is the first such report with *M. genitalium*. Thus far, *M. fermentens* is the only other mycoplasma that has been reported to differentiate THP-1 cells [61]. It is very likely that this ability can bestow additional advantages to *M. genitalium* while infecting the host, particularly in the modulation of host immune response which includes generation of ROS and NO. Thus, this property of *M. genitalium* may constitute an important virulence mechanism and identification of *M. genitalium* molecules that induce differentiation of THP-1 cells may provide additional insights into this mechanism. In *M. fermentens*, a 40 kDa surface lipoprotein has been implicated in the differentiation of monocytes by this species [61]. Since *M. genitalium* has an array of surface lipoproteins that have previously been implicated in induction of the host cell immune response and alteration of host cell signaling [14], [69], [70], it is likely that they also play a role in the differentiation of monocytes.

It is interesting to note that the *msrA* mutant strain of *M. genitalium* is deficient in inducing the proinflammatory cytokine TNF-α, the principal mediator of inflammatory response to infection. A previous study has indicated that *M. genitalium* is capable of inducing a variety of proinflammatory cytokines in human monocyte derived macrophages [59], thus it is likely that other proimflammatory cytokines may show similar responses to this strain. Lipid associated membrane proteins (LAMPs) of *M. genitalium* seems to be the major components that induce proinflammatory cytokines in host cells [14], [16], [70] in which the TLR2 receptor of the host cells play a major role [71]. Blocking of the TLR2 receptors resulted in reduced induction of TNF-α and IL-6 by LAMPs [70]. It has been suggested that pathogenic mycoplasmas adapt to different conditions in the host cells by changing the size and phase variations of LAMPs [72].

Our findings show that MsrA in *M. genitalium* is primarily localized in the cytoplasm. In contrast, some pathogenic bacteria like *A. actinomycetemcomitans* [33], *H. pylori* [35] and *N. gonorrhoeae* [73] have MsrA distributed between the cytosol and membrane, although the proportions vary. Among these species, only *A. actinomycetemcomitans* expresses MsrA as a single unit [31]. In *H. pylori* and *N. gonorrhoeae* MsrA is fused with either MsrB (MsrA-MsrB), or with Trx and MsrB (Trx-MsrRA-MsrB) [31]. It has been reported that only the cytosolic MsrA was active in *A. actinomycetemcomitans* [33] and only the membrane bound MsrA was active in *H. pylori* [35]. However, MsrA is a secretory protein in *A. actinomycetemcomitans*, *H. pylori* and *N. gonorrhoeae* [73], [74], [75]. The secreted MsrA should provide some benefit for these bacteria either in defending host derived ROS or in modulating the host cell machinery by reducing oxidized methionine in essential proteins.

In conclusion, it appears that the effect of MsrA on the virulence of *M. genitalium* is primarily due to modification of surface molecules that mediate interaction with host cells. Identification of the molecules affected by MsrA may provide important clues for the development of novel drugs against this pathogen.

## Supporting Information

Figure S1 **SDS-PAGE analysis of** ***M. genitalium*** **Cytosol and membrane fractions.** Cytosol and membrane fractions from *M. genitalium* strains, SDS-PAGE analysis and Western transfer were done as described in Materials and Methods section. The separated and transferred proteins were stained with Ponceau S. G37 and MS5 represent *M. genitalium* wild type and *msrA* mutant strains, respectively. W, M and C indicate whole, membrane and cytosol fractions of *M. genitalium*. Mr indicates molecular weight markers. Numbers on the left represent the sizes (kDa) of the markers.
(TIF)
