# Supplementary figures and images for: Methionine Sulfoxide Reductase A (MsrA) Deficient Mycoplasma genitalium Shows Decreased Interactions with Host Cells
Source: PLoS One. 2012 Apr 30;7(4):e36247. doi: 10.1371/journal.pone.0036247 (PMC3340341; doi:10.1371/journal.pone.0036247)

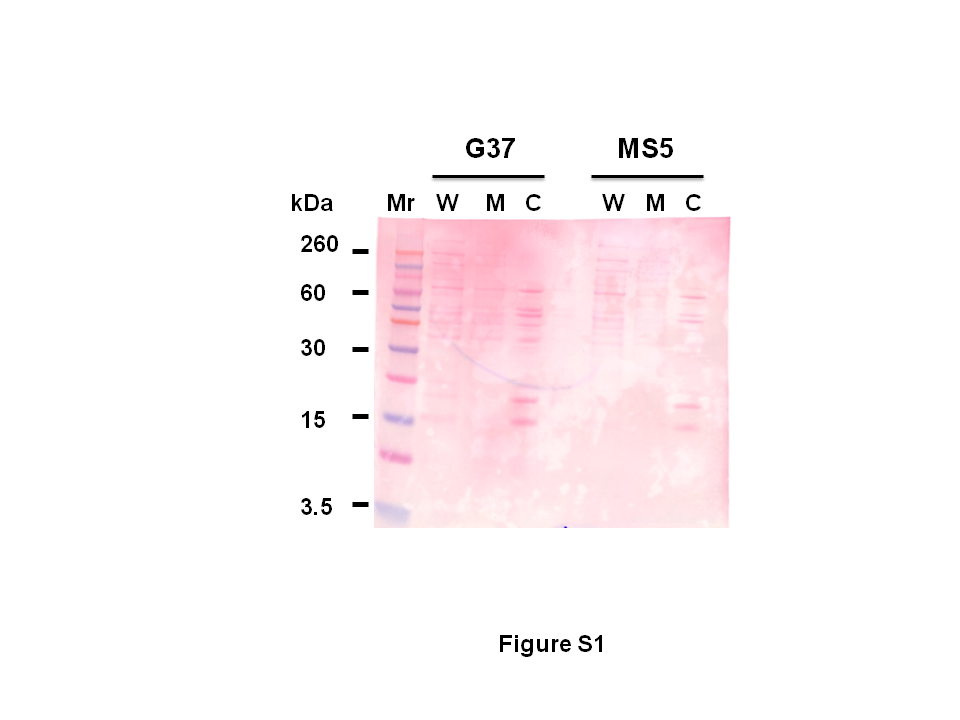

Supplement: Figure S1 — SDS-PAGE analysis of M. genitalium Cytosol and membrane fractions. Cytosol and membrane fractions from M. genitalium strains, SDS-PAGE analysis and Western transfer were done as described in Materials and Methods section. The separated and transferred proteins were stained with Ponceau S. G37 and MS5 represent M. genitalium wild type and msrA mutant strains, respectively. W, M and C indicate whole, membrane and cytosol fractions of M. genitalium. Mr indicates molecular weight markers. Numbers on the left represent the sizes (kDa) of the markers. (TIF) [file pone.0036247.s001.tif]
